# Hear Both Sides

**Published:** 1883-12

**Authors:** 


### HEAR BOTH SIDES.

We are not responsible for the opinions of contributors. There can be no fair discussion nor entire understanding of any subject, unless both sides be heard. Therefore we welcome all shades of opinion, asking only that they be intelligently and courteously presented. This journal aims to be the free unfettered organ of the profession at large, and if it accomplishes that it must frequently present articles which many will condemn. But, *Audi alteram partem*, is the only safe motto.

